# Renal and systemic complications in autosomal dominant hypocalcemia type 1: a case series from North East England

**DOI:** 10.1210/jendso/bvag205

**Published:** 2026-09-01

**Authors:** Deepika Manoharan, Edwin K S Wong, Holly Mabillard, Shalabh Srivastava, Simon H S Pearce, John A Sayer

**Affiliations:** Faculty of Medical Sciences, Biosciences Institute, Newcastle University, Newcastle Upon Tyne, Tyne and Wear, NE1 3BZ, UK; Renal Services, Newcastle Upon Tyne NHS Foundation Trust, Newcastle Upon Tyne, NE7 7DN, UK; Faculty of Medical Sciences, Biosciences Institute, Newcastle University, Newcastle Upon Tyne, Tyne and Wear, NE1 3BZ, UK; Renal Services, Newcastle Upon Tyne NHS Foundation Trust, Newcastle Upon Tyne, NE7 7DN, UK; Renal Services, Newcastle Upon Tyne NHS Foundation Trust, Newcastle Upon Tyne, NE7 7DN, UK; Faculty of Medical Sciences, Translational and Clinical Medicine Institute, Newcastle University, Newcastle Upon Tyne, NE4 2HH, UK; Faculty of Medical Sciences, Biosciences Institute, Newcastle University, Newcastle Upon Tyne, Tyne and Wear, NE1 3BZ, UK; Renal Services, Newcastle Upon Tyne NHS Foundation Trust, Newcastle Upon Tyne, NE7 7DN, UK; National Institute for Health Research, Newcastle Biomedical Research Centre, Newcastle Upon Tyne, NE4 5PL, UK

**Keywords:** autosomal dominant hypocalcemia type 1, calcium-sensing receptor, nephrocalcinosis, nephrolithiasis, chronic kidney disease, CASR variants

## Abstract

**Context:**

Autosomal dominant hypocalcemia type 1 (ADH1) is a rare genetic disorder caused by activating *CASR* variants, characterized by hypocalcemia, low/normal parathyroid hormone, and hypercalciuria. While managed with conservative calcium targets to prevent renal damage, the long-term systemic burden remains poorly defined.

**Objective:**

To describe a case series of adults with genetically confirmed AHD1.

**Methods:**

We reviewed 9 adults with genetically confirmed ADH1 in North East England (2005-2024). Clinical, biochemical, and imaging data were retrospectively analyzed from electronic records. Pathogenic *CASR* variants were identified via accredited genomic panels.

**Results:**

The cohort (n = 9; 5 female; age 24-57) was 89% familial, with all exhibiting persistent hypocalcemia and 66% presenting with hypomagnesemia. Renal involvement affected 78%, including nephrocalcinosis (56%), nephrolithiasis (67%), and chronic kidney disease (CKD), with 2 patients from 1 kindred progressing to kidney failure (KF) and death. Extrarenal features included seizures, intracranial calcifications, dental abnormalities, and neuropsychiatric symptoms, often persisting despite conservative strategies to limit calcium supplementation post diagnosis. No clear correlation was found between mean serum calcium levels and disease severity, highlighting substantial phenotypic variability.

**Conclusion:**

ADH1 is a multisystem disorder with substantial morbidity, including nephrocalcinosis, CKD, and a risk of KF despite cautious management. Other significant features include hypocalcemic seizures and intracranial calcification. These findings emphasize the necessity of early genetic diagnosis, family screening, and longitudinal renal surveillance. The high complication burden under conventional therapy underscores the need for targeted treatments, such as CaSR antagonists (calcilytics), to improve long-term outcomes.

Calcium homeostasis is tightly regulated through a feedback system involving the parathyroid glands, kidneys, bone, and intestine. The calcium-sensing receptor (CaSR), a class C G protein–coupled receptor, is present in most tissues, but is highly expressed in the parathyroid glands and kidney tubules, playing a key role in extracellular calcium regulation. The CaSR detects minute changes in the concentration of extracellular ionized calcium, and that of other cations such as magnesium, and regulates parathyroid hormone (PTH) secretion accordingly (1, 2). Activation of CaSR by rising calcium suppresses PTH synthesis and release, while simultaneously reducing renal tubular calcium reabsorption, both through direct CaSR signaling within the nephron and indirectly via reduced PTH activity at the distal tubule. The suppression of PTH also has a downstream negative effect on vitamin D hydroxylation to its activated form in the kidneys and vitamin D–induced calcium absorption in the gut (2).

Specifically, within the renal tubules the CaSR in the thick ascending limb of the Henle loop regulates urinary calcium excretion by responding to high serum calcium levels. When activated by hypercalcemia, the CaSR initiates the production of 20-hydroxyeicosatetraenoic acid (20-HETE), which inhibits the passive, paracellular reabsorption of calcium and sodium, thereby reducing the kidney's concentrating ability (3). In addition, activation of CaSR expressed on the luminal membrane of the cells of the inner medullary collecting duct reduces antidiuretic hormone-induced increases in water permeability. CaSR activation increases calcium excretion while preventing calcium stone formation through dilution. CaSR activation causes an arginine vasopressin resistance (4).

Pathogenic variants in *CASR* alter extracellular calcium sensing, causing inappropriate PTH release. Inactivating variants cause familial hypocalciuric hypercalcemia type 1 by decreasing sensitivity, resulting in hypercalcemia, while “activating” gain-of-function variants cause autosomal dominant hypocalcemia type 1 (ADH1) by increasing sensitivity, leading to hypocalcemia.

Thus, when the CaSR is overly sensitive to serum calcium, the consequence is a distinctive biochemical pattern: hypocalcemia and hypomagnesaemia with inappropriately low or normal PTH, hyperphosphatemia, and hypercalciuria (Supplementary Fig. S1) (5). More than 95% of variants are heterozygous missense changes that are pathogenic by causing inappropriate activation of the receptor (6-8).

Clinical manifestations of ADH1 vary in severity, from asymptomatic to severe symptomatic tetany, life-threatening hypocalcemic seizures, or laryngospasm (9, 10). Once treatment with activated vitamin D compounds or calcium is started in a patient with ADH1, the extracellular calcium concentration rises above the abnormally low calcium-sensing receptor set-point, leading to suppression of minute-to-minute PTH secretion. This effectively abrogates the acute regulatory mechanism for serum calcium levels, producing wider excursions in serum calcium than prior to treatment, which may lead to worse symptoms than before treatment. Neuropsychiatric symptoms, including depression, anxiety, and mood instability, are also frequently reported and often a feature of hypoparathyroidism (11). Cardiac complications may occur as well, with hypocalcemia predisposing to QTc prolongation and arrhythmias, particularly when accompanied by hypomagnesemia or hypokalemia (2, 11). Most important, chronic complications are well recognized and include nephrolithiasis and nephrocalcinosis, which increase the risk of chronic kidney disease (CKD) and progressive kidney failure (KF) (8, 10-12).

Ectopic intracranial calcifications can occur in one-third of cases, typically involving the basal ganglia, but may also extend to the thalamus, cerebellum, and other regions (10-12). Given the well-recognized ectopic calcifications in ADH1, aggressive correction of hypocalcemia with calcium and vitamin D supplementation can be harmful, worsening hypercalciuria and accelerating nephrocalcinosis, nephrolithiasis, and brain calcifications. Treatment goals should focus on maintaining a lower serum calcium (as symptoms allow) without excessive supplementation (2, 7, 11).

We report 9 genetically confirmed cases of ADH1 identified in North East England, detailing their genetic profiles as well as long-term biochemical, renal, and other health outcomes. This case series highlights the persistent morbidity and mortality seen in ADH1 patients despite careful management and low serum calcium targets, emphasizing the requirement for vigilant monitoring, individualized management strategies, and the need for new targeted treatments such as CASR antagonists (1, 8, 10).

## Materials and methods

### Study design and participants

This is a case series study of 9 patients with genetically confirmed ADH1 referred to and managed through the regional adult nephrology, endocrinology, and genetics services in North East England between 2005 and 2024. Patients older than 18 years with persistent hypocalcemia and inappropriately normal or low PTH levels were identified and subsequently confirmed to have a heterozygous pathogenic or likely pathogenic *CASR* variant through genetic testing. No patient who presented over age 18 with genetically confirmed ADH1 in North East England was omitted in the case series. Patients were consented to a national rare disease registry (RaDaR) (www.radar.org) (13).

### Genetic analysis

Following informed written consent, DNA was extracted from peripheral blood (EDTA sample) and sent to a national genomic laboratory for analysis for *CASR* variants. Gene panels R153 familial hypoparathyroidism or R256 nephrocalcinosis or nephrolithiasis were used. Variants were classified according to American College of Medical Genetics and Genomics criteria. Confirmed heterozygous pathogenic and likely pathogenic variants in *CASR* (NM_000388.4) (acting via gain of function) were considered diagnostic of ADH1. Segregation analysis of disease-causing variants was performed where possible.

### Data collection and statistical analysis

Demographics, genetic findings, biochemical measurements including adjusted serum calcium, serum magnesium, plasma intact PTH (automated intact PTH immunoassay), serum creatinine, calculation of estimated filtration glomerular rate (eGFR) using the CKD–Epidemiology Collaboration (CKD-EPI) creatinine formula, urine biochemistry, imaging (including ultrasound and computed tomography (CT) of the kidney, CT of the brain, echocardiogram), and clinical outcomes were all reviewed through NHS hospital trust electronic systems. Because methods for measuring intact PTH have evolved over the last two decades, we report low, normal, or high rather than absolute values. Both kidney involvement and systemic features were additionally assessed through documented history, latest imaging where available, and further consultation with the patient when possible. Given the small cohort size, data are presented descriptively as counts, percentages, and mean values.

## Results

**Figure 1:**
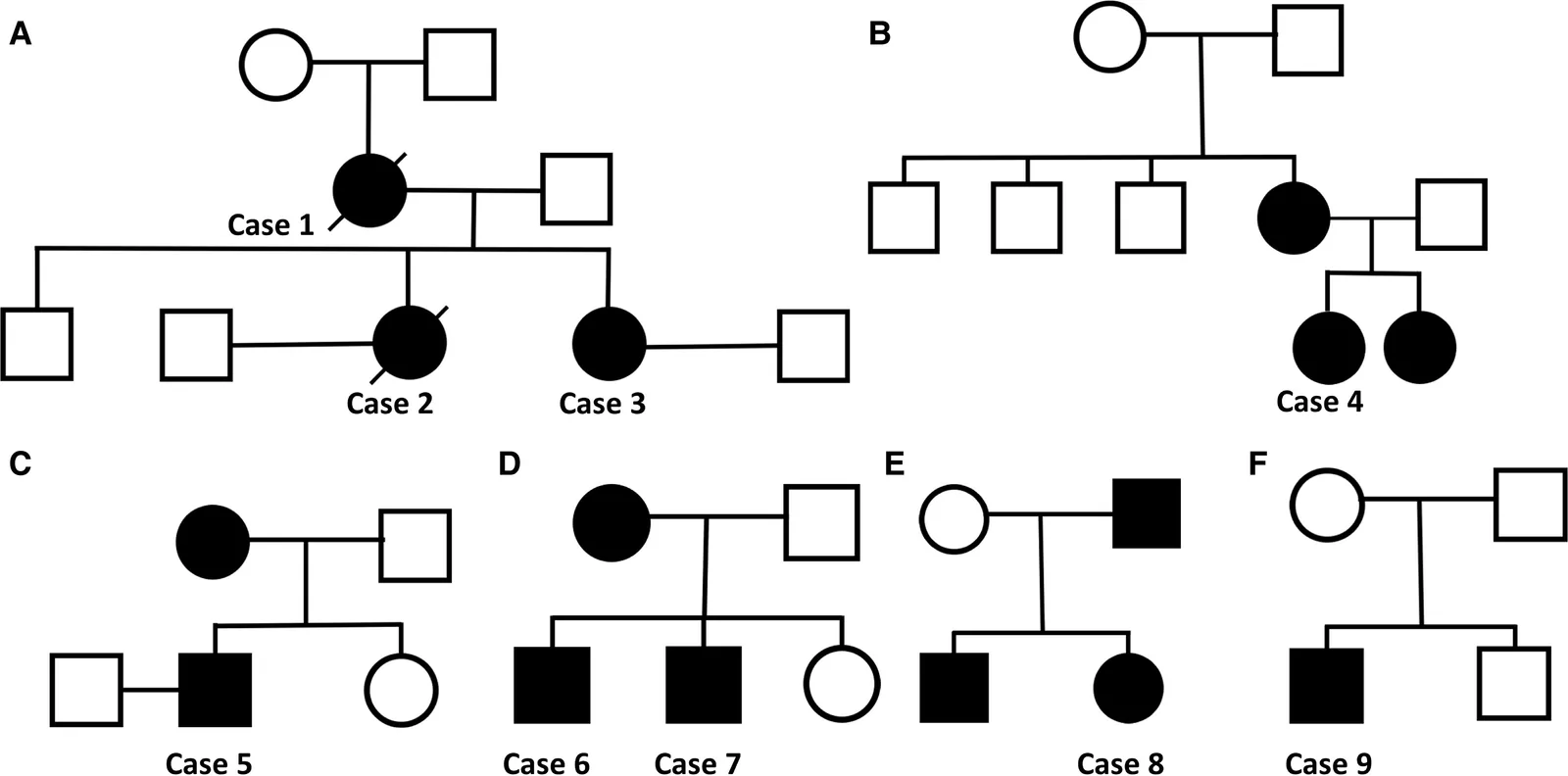
Pedigree diagrams for genetically proven autosomal dominant hypocalcemia type 1 cases. Pedigree diagrams for each family (1-6) showing cases 1 to 9. Circles indicate female family members, squares male family members, symbols with a slash representing deceased family members, and symbols with solid shade showing members who are affected.

Nine patients (female n = 5, male n = 4) were included in this study (Table 1). All were White British. Three related individuals across 2 generations from the same family (patient cases 1, 2, and 3) share the identical heterozygous *CASR* variant (c.354C > A; p.Asn118Lys). This family was described previously by Pearce et al 3 decades ago (12). Similarly, a pair of siblings (patient cases 6 and 7) from another family were heterozygous for the *CASR* variant (c.2053G > A; p.Gly685Ser). The remaining four patients carried individual heterozygous pathogenic or likely pathogenic missense alleles (see Table 1). Three of these individuals (patient cases 4, 5, and 8) had a clear family history of hypocalcemia and associated complications, but a formal molecular genetic diagnosis in affected family members was not made because they were either unavailable for testing or receiving care within other health-care trusts. With the assumption that a positive family history is equally significant as genetic confirmation, 8 (89%) of our patient cases were familial and 1 (11%) is likely a de novo mutation (Fig. 1).

**Table 1:** Demographics and clinical characteristics including the genetic mutation, biochemistry, family history, and complications in 9 patients with autosomal dominant hypocalcemia type 1

Family	Patient ID	Age, y	Sex	CASR variant, heterozygous	Current serum biochemistry (Ca/PTH/PO4)	Mean adjusted Ca, mmol/L*^a^*	Mean serum Mg, mmol/L*^b^*	Last eGFR, mL/min/1.73 m^2^	Family history	Renal complications	Other features and complications
1	1 (deceased)	59	F	c.354C > A; p.Asn118Lys	Ca↓ PTH↓ PO4↑	1.83	0.77	12	Yes	Nephrocalcinosis, nephrolithiasis, UTI, KF	Short stature, hypocalcemic seizures, brain calcification, depression, early-onset dementia, hypomagnesemia
1	2 (deceased)	32	F	c.354C > A; p.Asn118Lys	Ca↓ PTH↓ PO4↑	1.88	0.85	Dialysis-dependent	Yes	Nephrocalcinosis, nephrolithiasis, KF	Short stature, hypocalcemic seizures, brain calcification, HTN
1	3	35	F	c.354C > A; p.Asn118Lys	Ca↓ PTH→ PO4↑	1.52	0.66	47	Yes	Nephrocalcinosis, nephrolithiasis, UTI, CKD	Short stature, hypocalcemic seizures, brain calcification, HTN, hypomagnesemia, dental caries, hypoplastic teeth
2	4	25	F	c.2363T > G; p.Phe788Cys	Ca↓ PTH↓ PO4↑	1.59	0.6	>90	Yes	Nephrocalcinosis	Hypomagnesaemia, hypoplastic teeth, hypodontia
3	5	31	M	c.350A > G; p.Gln117Arg	Ca↓ PTH→ PO4↑	1.66	0.94	>90	Yes	None	Short stature, seizures, brain calcification, hypoplastic teeth, hypodontia
4	6	59	M	c.2053G > A; p.Gly685Ser	Ca↓ PTH→ PO4→	1.83	0.90	>90	Yes	Nephrolithiasis	HTN
4	7	49	M	c.2053G > A; p.Gly685Ser	Ca↓ PTH↓ PO4→	2.03	0.69	>90	Yes	None	None
5	8	38	F	c.416T > C; p.Ile139Thr	Ca↓ PTH↓ PO4→	2.1	0.67	>90	Yes	Nephrolithiasis	HTN, hypomagnesemia, dental caries, depression, anxiety
6	9	40	M	c.2495T > C; p.Phe832Ser	Ca↓ PTH↓ PO4→	1.26	0.62	>90	No	Nephrocalcinosis	Seizures, brain calcification, hypoplastic teeth, hypodontia

Reference sequence for CASR: NM_000388.4.

Abbreviations: Ca^2+^, calcium; CASR, calcium-sensing receptor; CKD, chronic kidney disease; eGFR, estimated filtration glomerular rate using CKD-EPI formula; F, female; HTN, hypertension; ID, identification; KF, kidney failure; M, male; PO_4-_, phosphate; PTH, parathyroid hormone; UTI, urinary tract infection.

^*a*^Mean adjusted serum calcium over at least 6 readings from current treatment regimens.

^*b*^Mean adjusted serum magnesium over at least 6 readings from current treatment regimes.

All patients (100%) demonstrated hypocalcemia with inappropriately low or normal PTH and two-thirds had significant hypomagnesemia (see Table 1). The mean serum magnesium of the cohort was 0.75 mmol/L. Serum phosphate was elevated in 5 cases (56%). The mean adjusted serum calcium across the cohort was 1.74 mmol/L (range, 1.26-2.10 mmol/L); however, 7 of the individuals were taking calcium or activated vitamin D at the time of the measurements, and pretreatment levels are therefore partially obscured.

**Figure 2:**
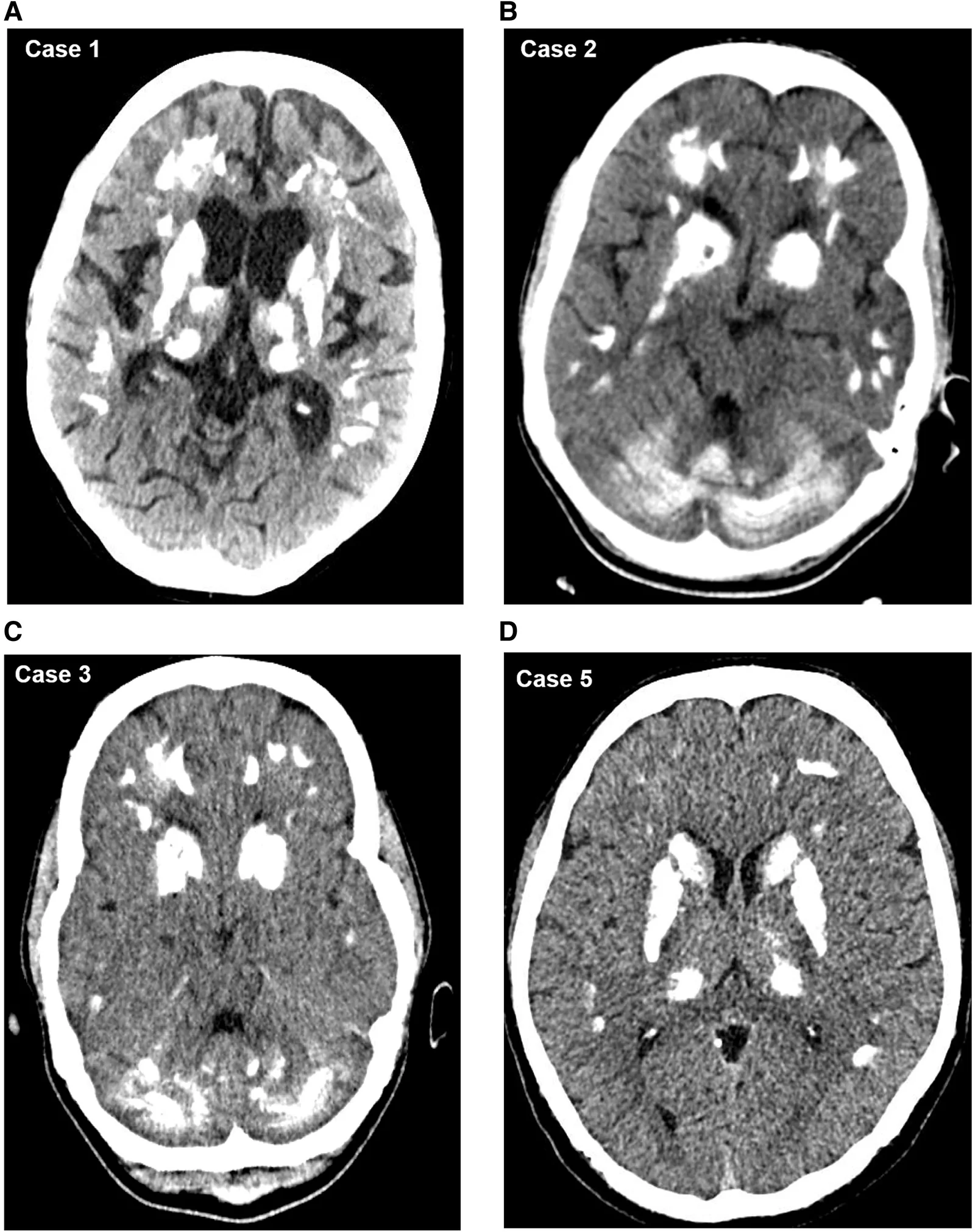
Intracranial calcification is a major phenotype of autosomal dominant hypocalcemia type 1 patients. Computed tomography imaging of brain (axial views) showing brain calcification in cases 1 to 3 (family 1) and 5 (family 3).

Renal involvement was prominent, affecting 7 (78%) patients, with nephrocalcinosis (n = 5), nephrolithiasis (n = 6), and recurrent urinary tract infections (UTIs) (n = 2) being main complications. Evidence of CKD was present in all 3 patients from 1 kindred (patient cases 1, 2, and 3), 2 of whom reached KF and subsequently died (aged 32 and 57 years, respectively). The third patient has stable stage 3 CKD. They also shared similar systemic manifestations (see Table 1), including short stature and brain calcifications (Fig. 2).

The extrarenal complications for all individuals (see Table 1) included seizures, brain calcification, teeth/dental abnormalities, and HTN. In all but one of the patient cases, calcium and vitamin D supplementation were either reduced or discontinued completely after a formal diagnosis and a lower serum calcium level (depending on patient tolerance and symptoms) was accepted as a physiological baseline. The overall outcomes were variable, demonstrating the phenotypic variability of ADH1 cases. A clinical summary of each case follows.

### Family 1 patient case 1

This female patient presented at age 21 years during her first pregnancy, with symptomatic hypocalcemia. She had symptomatic hypocalcemia (mean serum calcium 1.46 mmol/L at presentation) with episodic hypomagnesemia, persistent hypercalciuria, and inappropriately low PTH, complicated by a prolonged QTc on electrocardiogram and hypocalcemic seizures since childhood. She was also of short stature (height 1.49 m). She developed nephrocalcinosis and recurrent calcium oxalate nephrolithiasis over time, leading to multiple urological interventions, including ureteric stent insertions for hydronephrosis, and recurrent urinary tract infections, urosepsis, and acute kidney injury. She subsequently progressed to CKD stage 5. A molecular genetic diagnosis of ADH1 was confirmed (heterozygous likely pathogenic variant in *CASR* c.354C > A; p.Asn118Lys). With advancing age, she was noted to have intracranial calcifications (cerebellum, basal ganglia, and gray-white matter junction), associated with early cognitive decline, delirium, memory loss, tremor, and eventual dementia (see Fig. 2). Additional comorbidities included HTN, Raynaud phenomenon, headaches treated as migraines, low mood, and gastritis. She was maintained on alfacalcidol (1-α-hydroxyvitamin D_3_) 0.25 mcg alternative days. She experienced recurrent *Clostridium difficile* infections, attributed to repeated antibiotic exposure for urosepsis. Despite supportive management, she died at age 57 years from urosepsis and acute kidney injury. Her adjusted serum calcium (based on a mean of 6 readings) was 1.83 mmol/L and serum magnesium (based on a mean of 6 readings) was 0.77 mmol/L.

### Family 1 patient case 2

This female patient (the daughter of patient case 1) was diagnosed with hypocalcemia and hypocalcemic seizures during the neonatal period. She experienced lifelong complications including recurrent seizures, short stature (height 1.5 m), delayed puberty, hypertension (HTN), medullary nephrocalcinosis, nephrolithiasis, and progressive CKD. Her pregnancy was complicated by maternal HTN, warranting careful monitoring. She had a kidney biopsy at age 29 years for progressive CKD that showed chronic damage secondary to nephrocalcinosis and HTN. She was confirmed to have (inherited from her mother) the familial *CASR* variant (c.354C > A; p.Asn118Lys). She developed extensive intracranial calcifications (cerebral, thalamic, and cerebellar) (see Fig. 2) and ultimately progressed to KF by age 31 years, requiring hemodialysis followed by peritoneal dialysis while awaiting transplantation. Her cardiac evaluation demonstrated severe left ventricular systolic dysfunction but no evidence of calcification, and she died suddenly in the hospital at age 32 years following an unexpected cardiac arrest. She was not on any calcium or vitamin D supplements for at least 3 years prior to her death and had an adjusted serum calcium (based on a mean of 6 readings) of 1.88 mmol/L and a serum magnesium (based on a mean of 6 readings) of 0.85 mmol/L.

### Family 1 patient case 3

This 35-year-old woman (daughter of patient case 1 and sibling of patient case 2) was genetically diagnosed with ADH1 in early adulthood following genetic screening for familial *CASR* variant (c.354C > A; p.Asn118Lys). She was known to have asymptomatic hypocalcemia, hyperphosphatemia, reduced eGFR, and normal PTH levels. She also had short stature (height 1.34 m), nephrocalcinosis detected in her teenage years, and a right-sided renal staghorn calculus requiring percutaneous nephrolithotomy at age 16 years. She has also suffered from dental consequences, including multiple deep caries and hypoplastic teeth, necessitating multiple extractions. Her brain CT demonstrated significant cerebral and cerebellar calcifications by early adulthood (see Fig. 2). She had 3 successful pregnancies with the first and last complicated by preeclampsia. Additional comorbidities included HTN, aortic regurgitation, headaches (treated as migraines), and recurrent lower urinary tract symptoms with no positive urine cultures. She is currently asymptomatic and has an eGFR of 62 mL/min/1.73 m^2^ and has not been maintained on any calcium or vitamin D supplements, with a mean adjusted serum calcium of 1.52 mmol/L with a mean serum magnesium of 0.66 mmol/L.

### Family 2 patient case 4

This 25-year-old woman was confirmed to have ADH1 due to heterozygous pathogenic activating *CASR* variant (c.2363T > G; p.Phe788Cys) through genetic testing. She was initially noted to have severe hypocalcemia in infancy. Renal imaging in childhood revealed medullary nephrocalcinosis, though renal function remained normal. Dental issues, including poor development of the enamel and abnormal spacing, were corrected with braces. As an adult, she is largely asymptomatic, but can suffer from tingling in her hands when adjusted serum calcium levels drop below her baseline mean (<1.5 mmol/L). Her family history was significant for severe kidney disease, with her mother developing nephrocalcinosis and KF managed with hemodialysis and successful renal transplantation. Her sister also has hypocalcemia and frequently suffers from generalized seizures requiring hospital admissions. Her mother and sister are both likely affected by the same condition, although genetic testing has not been performed. She has not yet had brain imaging to assess for intracranial calcifications. She has very poor compliance in attending medical appointments and monitoring blood tests for serum calcium levels. She is maintained on alfacalcidol 0.5 mcg daily and calcium supplementation (2000 mg calcium per day) with a caution of not increasing her adjusted calcium levels more than 1.6 mmol/L and a mean serum magnesium of 0.60 mmol/L. At present she has preserved kidney function, with an eGFR greater than 90 mL/min/1.73 m^2^.

### Family 3 patient case 5

This patient is a 31-year-old man with a history of febrile convulsions in early childhood and was noted to have hypocalcemia and hyperphosphatemia with normal PTH levels. He was diagnosed with ADH1 due to likely pathogenic activating mutation of the *CASR* gene (c.350A > G; p.Gln117Arg) introducing an amino acid with a gain of a positive charge. Other clinical features include short stature (1.6 m), hypodontia with lack of dental enamel, and delayed eruption of teeth, although he has been grossly asymptomatic since adulthood. He has no obvious renal complications. Brain imaging confirmed dense symmetrical calcifications (cerebrum, cerebellum, and thalami) (see Fig. 2) with no obvious neurological or cognitive deficits on examination, although he suffers from stuttering and describes learning difficulties. Family history was notable for hypoparathyroidism and convulsions in his mother from infancy who currently continues on lifelong calcium supplementation and also has similar dental enamel defects and a short stature. He is maintained on Adcal D3 tablets twice a day (1200 mg calcium/day) and alfacalcidol 0.25 mcg daily. His mean serum calcium is 1.66 mmol/L, with a mean serum magnesium of 0.94 mmol/L. His renal function is preserved, with an eGFR greater than 90 mL/min/1.73 m^2^.

### Family 4 patient case 6

This 59-year-old man suffered from recurrent neuromuscular and neurological manifestations, including paresthesia, muscle cramps, and seizures attributed to hypocalcemia since birth. Biochemical profiles repeatedly demonstrated low serum calcium with inappropriately low to normal PTH levels, and he was maintained on calcium and vitamin D supplements for the vast majority of his life. Doses used were typically calcitriol 5 mcg/day (1,25-dihydroxyvitamin D_3_) and calcium supplements 12 000 mg/day to maintain a serum calcium of 1.7 to 1.8 mmol/L. He also has bilateral nephrolithiasis confirmed on CT imaging. Although it was suspected that he likely had CASR abnormality, genetic testing showing a likely pathogenic activating mutation of the *CASR* gene (c.2053G > A; p.Gly685Ser) was identified only at age 57. His mother and brother are also affected by the same condition. Renal function has remained normal (eGFR >90 mL/min/1.73 m^2^), and there is no evidence of nephrocalcinosis. Neuroimaging had not been performed. He had other comorbidities including HTN, chronic obstructive pulmonary disease, and chronic diarrhea. He is now maintained on reduced doses of active vitamin D (calcitriol 2 mcg once a day [1,25-dihydroxyvitamin D_3_] and calcium supplements [10 000 mg/day]) but continues to have severe, persistent muscle cramps with a mean serum calcium concentration of 1.83 mmol/L with a mean serum magnesium of 0.9 mmol/L.

### Family 4 patient case 7

This 49-year-old man (brother of patient case 6) had documented hypocalcemia from birth and a history of recurrent seizures that started at age 20 years but with normal electroencephalogram and a normal magnetic resonance imaging of the head. He was found to have an activating mutation of the *CASR* gene (c.2053G > A; p.Gly685Ser). His recurrent seizures are most likely associated with severe hypocalcemia. He has no renal complications, and recent brain imaging did not show ectopic calcification. He is currently maintained with alfacalcidol 0.5 mcg daily, and calcium carbonate 5 g twice daily (4000 mg calcium/day) and has a mean serum calcium level of 2.03 mmol/L with a mean serum magnesium of 0.69 mmol/L. His renal function is normal, with an eGFR greater than 90 mL/min/1.73 m^2^.

### Family 5 patient case 8

This is a 38-year-old woman with hypocalcemia secondary to a likely pathogenic activating mutation of the *CASR* gene (c.416T > C; p.Ile139Thr). She suffers from recurrent neuromuscular symptoms, including cramps and intermittent tingling since childhood. She has poor dentition with multiple caries, and a history of HTN and nephrolithiasis, but with no nephrocalcinosis identified on kidney imaging, and preserved kidney function (eGFR >90 mL/min/1.73 m^2^). Her medical history also includes depression, anxiety, diabetes mellitus type 2 treated with metformin since age 36 years, and chronic diarrhea. Family history was notable, with both her father and brother having hypocalcemia. She is maintained on treatment with alfacalcidol 0.75 mcg and calcium carbonate–colecalciferol D_3_ two tablets daily (1000 mg calcium and 800 U of colecalciferol/day) with a mean serum calcium of 2.1 mmol/L and a mean serum magnesium of 0.67 mmol/L.

### Family 6 patient case 9

This 40-year-old man was born with cerebral palsy affecting all 4 limbs, with intermittent left-sided flexor spasms, mild spastic weakness, and higher-level cognitive difficulties. He was admitted to the neonatal unit within hours of birth following seizures and was found to have hypocalcemia and hypomagnesemia. Although his PTH levels were normal, he was diagnosed clinically with hypoparathyroidism and treated with calcium and vitamin D. There were no known affected family members, and no known family history of calcium disorders, although his mother was adopted so more distant family history was unavailable. During early childhood he continued on calcium supplementation, but nephrocalcinosis was detected prompting a more detailed assessment when he was age 10 years. A CaSR abnormality was suspected and confirmed with the identification of a likely pathogenic activating mutation of the *CASR* gene (c.2495T > C; p.Phe832Ser). He has hypodontia with enamel defects, and progressive ectopic calcification involving the kidneys, liver, brain, and soft tissues. Despite his nephrocalcinosis, renal function remains normal (eGFR >90 mL/min/1.73 m^2^). He is maintained on calcium carbonate (total of 2000 mg calcium/day), and alfacalcidol 0.5 mcg daily with a mean serum calcium of 1.26 mmol/L and a mean serum magnesium of 0.62 mmol/L.

## Discussion

This case series of 9 genetically confirmed ADH1 patients provides a detailed longitudinal perspective on the renal and multisystem burden associated with heterozygous activating variants in *CASR*. ADH1 usually presents with hypocalcemic symptoms that vary widely in severity, from asymptomatic disease to paresthesia, carpopedal spasm, muscle cramps, tetany, laryngospasm, and seizures. The kidney phenotype is central to features of ADH1. Hypercalciuria is often present and leads to nephrocalcinosis, nephrolithiasis, renal impairment, and other calcification complications over time. Neurologic and ectopic calcification phenotypes extend beyond acute hypocalcemic symptoms. Basal ganglia or intracranial calcifications are frequently seen.

Our findings reinforce that ADH1 is not a benign biochemical disorder, but a chronic, multisystem disease characterized by persistent hypocalcemia, hypercalciuria, and progressive organ complications, particularly affecting the kidneys and central nervous system. Consistent with previous findings, our study suggests that this condition may be underdiagnosed and subject to considerable diagnostic delays (14).

### Renal morbidity and risk of progressive kidney disease

Renal involvement was the most prominent complication in our cohort, affecting 78% of patients, with nephrocalcinosis, nephrolithiasis, recurrent UTIs, CKD, and KF observed. We detail 2 individuals that progressed to KF at relatively young ages and note KF in the affected mother of another patient, highlighting the potential for severe long-term renal sequelae in ADH1. These observations are consistent with prior reports demonstrating that hypercalciuria driven by CaSR overactivation leads to increased urinary calcium excretion and a high burden of renal calcification (8, 11). The CaSR is highly expressed in the thick ascending limb and distal nephron, where gain-of-function variants reduce tubular calcium reabsorption, predisposing to nephrocalcinosis and nephrolithiasis despite systemic hypocalcemia (1). Hypercalciuria will be exacerbated by overcorrection of serum calcium in ADH1 patients (10).

Previous case series and reviews have similarly described renal calcification as a hallmark complication of ADH1, with long-term risks of CKD and kidney impairment (11). Our series provides examples where significant renal morbidity exists and was, in some cases, likely complicated by calcium supplementation. The clustering of KF within at least one kindred in our cohort also raises the possibility of intrafamilial modifiers, including genetic background, environmental factors, or treatment exposure, influencing renal outcomes.

In ADH1, conventional treatment with calcium and active vitamin D may further suppress PTH secretion and increase urinary calcium excretion. Because PTH is a key regulator of acute calcium homeostasis and renal activation of vitamin D, escalating therapy to correct hypocalcemia can worsen hypercalciuria, thereby increasing the risk of nephrocalcinosis, nephrolithiasis, and CKD. This therapeutic dilemma underscores the need for disease-specific treatments that target the underlying activating CaSR defect (10, 11). Some patients report thirst and polyuria as serum calcium approaches the normal range, potentially reflecting renal CaSR activation and a urinary-concentrating defect analogous to that seen in hypercalcemia-induced nephrogenic diabetes insipidus. Magnesium replacement may be required for some ADH1 patients. For management decisions, treatment should be personalized based on serum magnesium, urinary losses, kidney function, symptoms, and concomitant therapies.

### Multisystem phenotype beyond classical biochemical abnormalities

Although ADH1 is classically defined by hypocalcemia with low or inappropriately normal PTH levels, our data emphasize its broader multisystem phenotype. Neurological manifestations, including hypocalcemic seizures and extensive intracranial calcifications, were frequent and align with previous reports describing basal ganglia and cerebellar calcifications in up to one-third of affected individuals (11, 15). The presence of neuropsychiatric symptoms and cognitive decline in some patients further supports the concept that chronic hypocalcemia and ectopic calcification contribute to neurological morbidity over time.

Dental abnormalities, including enamel hypoplasia and hypodontia, were also prominent in our cohort but remain underrecognized in the literature. While these features have been sporadically reported in CaSR-related disorders, they are not routinely emphasized as core manifestations. Similarly, HTN and short stature were observed in multiple patients. HTN is not a typical disease manifestation of ADH1, but it is likely that patients with CKD developed elevated blood pressure as a consequence of this. Indeed, in the severe “Bartter like” cases you might expect low blood pressure through renal salt-wasting. Short stature is more commonly associated with ADH2 (*GNA11* variants) (16) but our observations suggest that growth and developmental effects may also occur in ADH1, possibly reflecting chronic biochemical disturbance or shared downstream signaling pathways. ADH1 likely causes a low-turnover bone phenotype with possible microdamage-related fragility, but the frequency of fractures and the full spectrum of human skeletal complications remain uncertain. In general, patients with hypoparathyroidism have preserved or even high bone mineral density on dual-energy x-ray absorptiometry scanning. Collectively, these findings support the classification of ADH1 as a multisystem disorder rather than an isolated endocrine abnormality.

### Phenotypic variability and genotype-phenotype considerations

Marked phenotypic heterogeneity was evident across our cohort, even among individuals harboring the same *CASR* variant. Within a single family, renal outcomes ranged from stable stage 3 CKD to stage 5 CKD despite similar serum calcium biochemistry, suggesting that additional factors, including aggressiveness of calcium replacement and patient adherence to treatment, and episodes of urosepsis, contributed to the differences in disease severity. The overall phenotypic variability mirrors prior observations that genotype-phenotype correlations in ADH1 are inconsistent and that disease severity cannot be reliably predicted by serum calcium levels alone (6, 11). Additional modifiers such as adherence, cumulative calcium and vitamin D exposure, and individual renal susceptibility may contribute to outcome variability.

### Effect and limitations of conventional therapy

Management of ADH1 remains challenging. Traditional treatment strategies using calcium and vitamin D replacement aim to alleviate symptomatic hypocalcemia but often exacerbate hypercalciuria, thereby accelerating nephrocalcinosis and nephrolithiasis (10). In our cohort, supplementation was reduced or discontinued in most patients after molecular genetic diagnosis, and lower serum calcium targets were accepted as physiological baselines. Active vitamin D analogues were also avoided as much as possible; despite this conservative approach, renal calcification and kidney impairment remained common, highlighting the limitations of conventional therapy. These findings align with systematic reviews indicating that aggressive calcium correction may worsen renal outcomes without fully preventing symptoms (11).

Parathyroid gland (PTG) transplantation has been explored as a therapeutic strategy for severe hypoparathyroidism, including in the context of kidney disease. In this setting, simultaneous kidney-parathyroid transplantation and delayed PTG allotransplantation have both demonstrated biological feasibility, with several cases showing restoration of endogenous PTH secretion, improved calcium homeostasis, and, in some instances, independence from calcium and active vitamin D supplementation. However, outcomes are variable, with inconsistent long-term graft function, uncertain durability, and a lack of standardization in surgical technique, tissue dose, and immunosuppression protocols. Importantly, there are no published cases specifically addressing ADH1 rather than intrinsic gland failure (17, 18). Thus, while PTG transplantation offers proof of principle for physiological hormone replacement in true hypoparathyroid states, its role in ADH1 remains theoretical and uncertain.

### Implications for emerging targeted therapies

The persistent morbidity observed in this series underscores the need for disease-modifying therapies.

PTH injections and infusions can improve serum calcium in ADH1, but evidence remains limited and renal benefits vary. Improved biochemical control can be achieved with PTH rather than conventional calcium plus active vitamin D in some refractory patients, especially with continuous subcutaneous PTH(1-34) infusion (19), but the evidence base is still mostly small case series and observational cohorts rather than randomized ADH1 trials (20). Calcilytics (CaSR antagonists) represent a new promising targeted approach (21). Early clinical studies of encaleret (CALIBRATE: A Phase 3, Randomized, Open-Label Study Evaluating the Efficacy and Safety of Encaleret Compared to Standard of Care in Participants With Autosomal Dominant Hypocalcemia Type 1 [ADH1]) (ClinicalTrials.gov ID NCT05680818) in adults with ADH1 has demonstrated correction of hypocalcemia alongside reductions in urinary calcium excretion, suggesting potential renal protective effects (10, 21-23) . Such therapies may improve patient compliance, reduce reliance on high-dose supplementation, and mitigate long-term renal damage, particularly if introduced early in the disease course. An ongoing study in children with ADH1 is currently actively recruiting (A Phase 2/3, Multicenter, Single-Arm, Open-Label Study Evaluating the Pharmacokinetics, Efficacy, and Safety of Encaleret in Pediatric Participants With Autosomal Dominant Hypocalcemia Type 1 [ADH1]) ClinicalTrials.gov ID NCT07080385).

### Importance of genetic diagnosis and family screening

Our series also highlights the diagnostic delays that commonly occur in ADH1 (9), with several patients initially mislabeled as having idiopathic hypoparathyroidism for decades before genetic confirmation. Early molecular diagnosis is critical, as it directly influences management strategy, prevents overtreatment with calcium and vitamin D, and enables appropriate family screening. The high proportion of familial cases (89%) in our cohort supports routine genetic testing in individuals with unexplained hypocalcemia and low or normal PTH levels, as recommended in recent clinical practice reviews (10). Genetic identification also has implications for prenatal counseling and risk stratification in affected families.

### Strengths and limitations

The strengths of this study include genetically confirmed diagnoses, detailed longitudinal clinical data, and comprehensive assessment of renal and systemic complications within a well-defined regional cohort. However, limitations include the small sample size inherent to rare disease studies, retrospective data collection, and incomplete imaging or family genetic testing in some cases. Additionally, the cohort was ethnically homogeneous, which may limit generalizability.

In summary, this case series demonstrates that ADH1 is associated with substantial long-term kidney and multisystem morbidity, including a significant risk of nephrocalcinosis, nephrolithiasis, CKD, and KF, even with cautious biochemical management of hypocalcemia. Extrarenal features include hypocalcemic seizures and intracranial calcification. The discordance between biochemical control and clinical outcomes highlights the intrinsic renal effects of CaSR overactivation and the need for early diagnosis, vigilant symptom and signs surveillance, and personalized management strategies. Future research should focus on longitudinal multicenter cohorts, genotype-phenotype modifiers, and clinical trials of calcilytics to determine whether targeted CaSR antagonism can alter the natural history of renal disease in ADH1. A broader recognition of ADH1 may prevent misdiagnosis, guide family screening, and ultimately improve renal outcomes for affected patients.

## Data Availability

Data sharing is not applicable to this article as no datasets were generated or analyzed during the current study.

## Current affiliation

D.M. and S.S. is South Tyneside and Sunderland NHS Foundation Trust, Sunderland, UK.

## Abbreviations

ADH1: autosomal dominant hypocalcemia type 1
CASR: calcium-sensing receptor
CKD: chronic kidney disease
CT: computed tomography
eGFR: estimated filtration glomerular rate
HTN: hypertension
KF: kidney failure
PTG: parathyroid gland
PTH: parathyroid hormone
UTI: urinary tract infection
